# Rheological, Viscoelastic, and Dynamic Mechanical Behavior of ZnO-Filled and MA-Modified PP/SEBS Filaments for Fused Filament Fabrication

**DOI:** 10.1021/acsomega.6c06409

**Published:** 2026-09-24

**Authors:** Chiara das Dores do Nascimento, Everton Granemann Souza, Marcel Luiz Basso, Patricia Oliveira Schmitt, Andre Lamounier Caixeta, Eduarda Vieira Silva, Fabricio Celso, Cesar Aguzzoli, Lincoln Audrew Cordeiro, Marlon Bender Bueno Rodrigues, Arthur Lima Capriolli, Juliano Marini, Andre Luiz Missio, Amanda Dantas de Oliveira

**Affiliations:** † Graduate Program in Materials Science and Engineering (PPGCEM), 37902 Federal University of Pelotas (UFPel), Pelotas, RS 96010-610, Brazil; ‡ Graduate Program in Sciences and Technologies in Education (PPGCITED), Federal Institute of Education, Science and Technology of Rio Grande do Sul (IFSul), Pelotas, RS 96060-290, Brazil; ¶ Graduate Program in Electronics and Computer Engineering (MEEC), 67714 Catholic University of Pelotas (UCPel), Pelotas, RS 96015-560, Brazil; § Graduate Program in Materials Science (PPGCIMAT), Federal University of Rio Grande do Sul (UFRGS), Porto Alegre, RS 91501-970, Brazil; ∥ Graduate Program in Materials Science and Engineering (PPGMAT), University of Caxias do Sul (UCS), Caxias do Sul, RS 95070-560, Brazil; ⊥ Department of Materials Science, Federal University of Rio Grande do Sul (UFRGS), Porto Alegre, RS 91501-970, Brazil; # Graduate Program in Materials Science and Engineering (PPGMAT), Federal University of São Carlos (UFSCar), São Carlos, SP 13565-905, Brazil

## Abstract

Immiscible polyolefin–elastomer blends often exhibit limited impact resistance and unstable melt-flow behavior due to weak interfacial adhesion and inefficient stress transfer between phases. These limitations are relevant for fused filament fabrication (FFF), where filaments must withstand feeding, extrusion, deposition, and service loading. In this work, PP/SEBS (70/30 wt %) blends modified with maleic anhydride (MA) and ZnO nanoparticles were investigated to determine the influence of interphase engineering on the rheological, viscoelastic, dynamic mechanical, and FFF performance of PP/SEBS filaments. Four formulations (PP/SEBS, PP/SEBS-1%ZnO, PP/SEBS-3%MA, and PP/SEBS-1%ZnO-3%MA) were produced as 1.75 mm filaments and characterized by rotational rheology, DMA, tensile and notched Izod impact tests, SEM, and FTIR. All systems exhibited shear-thinning behavior, while the PP/SEBS-1%ZnO-3%MA formulation showed the most balanced melt response, combining reduced viscosity with adequate viscoelasticity. SEM and DMA analyses suggested improved interphase stability and more efficient viscoelastic dissipation in MA-containing systems. The PP/SEBS-1%ZnO-3%MA blend achieved the highest impact strength (46.86 ± 0.73 kJ m^–2^), approximately 60% higher than PP/SEBS, while maintaining tensile performance. Preliminary FFF trials demonstrated stable deposition and good dimensional reproducibility. These results demonstrate that MA/ZnO interphase engineering is an effective strategy for improving rheological stability and impact reliability in PP/SEBS filaments for FFF applications.

## Graphical abstract

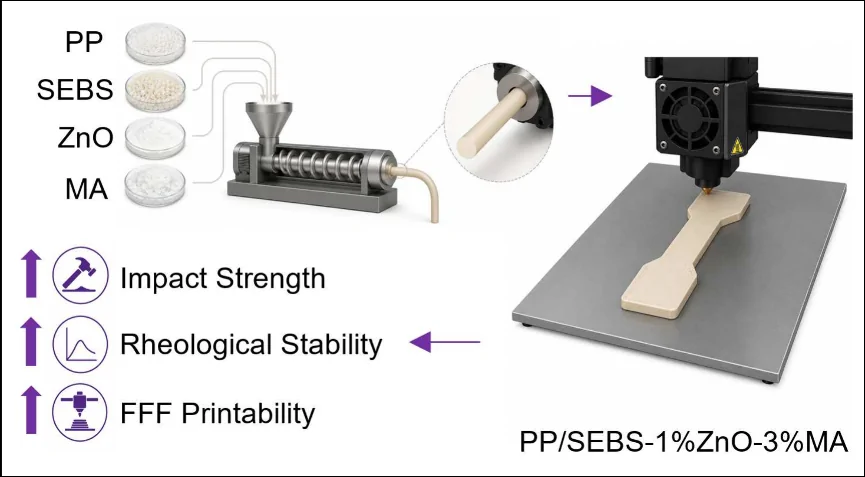

## 1 Introduction

Additive manufacturing based on material extrusion, commonly implemented as fused filament fabrication (FFF), has emerged as a versatile technology for producing complex geometries with short lead times, design flexibility and potential for decentralized production.^1–3^ Since the final performance of FFF components depends on both the properties of the filament and the quality of its deposition during printing,^4^ the development of advanced filament materials remains an active area of research.

However, despite these manufacturing advantages, the mechanical and thermomechanical performance of polymeric filaments remains a critical bottleneck for extending FFF toward truly structural applications. Compared with commonly used FFF polymers such as PLA and PET-G, polypropylene (PP) offers a distinct combination of low density, chemical resistance, ductility, and fatigue tolerance, making it attractive for lightweight functional components subjected to repeated deformation or impact loading. Nevertheless, PP-based systems still face important processing and performance limitations, including volumetric shrinkage, weak interlayer adhesion, limited interfacial toughness, and dimensional instability during printing.^1,2,5^

The incorporation of thermoplastic elastomers, particularly styrene–ethylene–butylene–styrene (SEBS), has been widely investigated as an effective strategy to enhance ductility, dimensional stability, and energy absorption in PP-based systems.^6–13^ Elastomer-modified PP blends can exhibit reduced effective crystallinity, improved impact resistance and more favorable melt rheology, all of which are desirable for extrusion-based additive manufacturing.^6–8^ Nevertheless, PP and SEBS are intrinsically immiscible, and their polarity mismatch leads to weak interfacial adhesion, limited stress transfer and premature interfacial debonding under dynamic loading conditions.^11–13^ As a consequence, the toughening efficiency of the elastomeric phase is strongly dependent on interphase engineering and morphology control.

To overcome these limitations, compatibilization strategies based on maleic anhydride (MA) have been extensively employed to improve the interphase of immiscible PP-based blends, most commonly through grafted compatibilizers such as PP-*g*-MA and SEBS-*g*-MA.^14–18^ These compatibilization strategies reduce phase separation and enhance stress transfer between otherwise poorly compatible phases through the presence of polar anhydride functionalities. In the present work, the direct incorporation of free MA during melt processing is investigated as an alternative strategy for modifying the blend interphase without employing pregrafted compatibilizers. Such interphase modifications are directly associated with processing–microstructure–toughness relationships, where cavitation, shear yielding, crack deflection and localized plastic deformation become strongly dependent on interphase integrity and strain-rate-sensitive energy-dissipation mechanisms.^13,19–23^

In parallel, inorganic nanoparticles such as CaCO_3_, graphene, CaTiO_3_, and ZnO have been incorporated into polyolefin systems as rigid reinforcing phases and nucleating agents capable of modifying crystallization behavior, viscoelastic response, and fracture resistance.^3,24,25^ Among these fillers, ZnO is particularly attractive because, besides contributing to stiffness and thermal stability, it also provides functional properties such as photocatalytic activity and antimicrobial behavior.^26,27^ More recently, hybrid systems combining compatibilization strategies and inorganic reinforcements have shown promising results in PP composites reinforced with mineral fillers, basalt fibers and lignocellulosic materials.^10,28–31^

A recent study on PP/SEBS/ZnO/MA systems demonstrated that the simultaneous incorporation of ZnO and MA improves morphology refinement, crystallinity, thermal stability, tensile performance and preliminary FFF processability of PP-based blends intended for additive manufacturing applications.^32^ However, despite these advances, the viscoelastic and rheological mechanisms governing melt-flow stability, energy dissipation and dynamic mechanical behavior in these systems remain poorly understood. In particular, previous studies have predominantly focused on static structural and tensile characterization, whereas the relationships between interfacial morphology, melt rheology, impact response and flow behavior under processing-relevant conditions have not yet been systematically investigated.

To systematically decouple the roles of MA addition and nanoparticle reinforcement, four formulations were designed in this work as a controlled experimental set: a reference immiscible blend (PP/SEBS), a nanoparticle-modified system without interfacial coupling (PP/SEBS-1%ZnO), an MA-containing blend without inorganic reinforcement (PP/SEBS-3%MA), and a hybrid system combining both strategies (PP/SEBS-1%ZnO-3%MA).

The central hypothesis of this work is that the simultaneous incorporation of MA and ZnO produces a synergistic interphase effect capable of improving stress transfer, stabilizing localized deformation mechanisms and promoting more efficient viscoelastic energy dissipation under dynamic loading. In this context, MA is expected to promote interphase modification and morphology refinement, whereas ZnO may contribute to localized stress redistribution and viscoelastic reinforcement. As a result, the hybrid system is expected to exhibit a more balanced combination of melt-flow behavior, viscoelastic integrity and impact resistance than systems containing only compatibilizer or nanoparticles individually.

Accordingly, this work investigates the combined influence of MA and ZnO on the rheological, dynamic mechanical and fracture behavior of PP/SEBS blends processed as filaments for FFF applications. Particular emphasis is placed on understanding how interphase-controlled mechanisms govern melt rheology, viscoelastic dissipation and impact reliability in immiscible polyolefin-based systems.

## 2 Materials and Methods

### 2.1 Materials

Polypropylene (PP), commercially known as PH 0952 resin, with a melt flow index of 8 g/10 min (ASTM D1238, Condition L; Braskem S.A., Triunfo, RS, Brazil), and SEBS (grade G1643, a nonoil-extended thermoplastic elastomer (TPE) of the Enhanced Rubber Segment (ERS) family; linear triblock copolymer; density 0.91 g/cm^3^; Shore A hardness 75; styrene content ∼ 30 wt %; Kraton Polymers, Houston, TX, USA) were used as the base blend components. Maleic anhydride (MA; C_4_H_2_O_3_ purity ≥ 99%; Sigma-Aldrich, St. Louis, MO, USA) was incorporated as a low-molecular-weight polar additive intended to modify the blend interphase. Zinc oxide (ZnO) nanoparticles (powder; average particle size 30–50 nm; purity ≥ 99.5%; Sigma-Aldrich, St. Louis, MO, USA) were used as a functional filler. All materials were used as received, without further purification.

### 2.2 Blend Preparation

The base formulation comprised 70 wt % PP and 30 wt % SEBS. Starting from this control blend, MA (3 wt %) and/or ZnO nanoparticles (1 wt %) were incorporated to modify the blend interphase and provide functional reinforcement, yielding four formulations, as summarized in Table 1.

**Table 1:** Compositional Design of PP/SEBS-Based Blends Containing ZnO Nanoparticles and MA^a^

sample	description	PP (wt %)	SEBS (wt %)	MA (wt %)	ZnO (wt %)
PP/SEBS	neat PP/SEBS blend	70.0	30.0	0.0	0.0
PP/SEBS-1%ZnO	PP/SEBS with 1 wt % ZnO nanoparticles	69.0	30.0	0.0	1.0
PP/SEBS-3%MA	PP/SEBS with 3 wt % MA	67.0	30.0	3.0	0.0
PP/SEBS-1%ZnO-3%MA	PP/SEBS with 3 wt % MA and 1 wt % ZnO	66.0	30.0	3.0	1.0

a Compositions are expressed in weight percent (w/w) relative to the total formulation.

### 2.3 Filament Extrusion Processing

The formulations were compounded using a bench-scale single-screw mini-extruder (E-Lit Engenharia Linear, São Carlos, SP, Brazil) equipped with an anodized aluminum frame, a 20 mm screw, and an L/D ratio of 25:1. Interchangeable dies (1.0–1.8 mm) were available. A 1.8 mm die was selected, and the filament diameter was measured at 30 equally spaced positions along 1 m of filament, resulting in a final diameter of 1.75 ± 0.05 mm, suitable for FFF.

The temperature profile along the barrel was maintained at 230 °C, while the die temperature was also set to 230 °C. Screw speed was monitored by a tachometer-based setup (Minipa, Model MDT-2238A, São Paulo, SP, Brazil) over 60 rpm and an empirical calibration curve was obtained from $v=\frac{\Delta s}{\Delta t}$ , where *v* is the linear speed of the moving filament line, Δ*s* is the distance traveled by the filament during the measurement interval, and Δ*t* is the elapsed time. The extruded filaments were air-cooled at room temperature using a simple desktop fan and collected under continuous monitoring to ensure dimensional uniformity and process stability.

### 2.4 FFF Printing Conditions

FFF experiments were carried out using a Creality K1Max 3D printer and processed with Creality Print slicing software using a customized printing profile. The filament diameter and material density were set to 1.75 mm and 0.91 g cm^–3^, respectively.

Printing was performed using a 0.4 mm brass nozzle at an extrusion temperature of 240 °C for both the initial and subsequent layers. The build plate and chamber temperatures were maintained at 100 and 50 °C, respectively. A textured PEI build surface was employed throughout the printing process.

The flow ratio was adjusted to 1.20, while the maximum volumetric flow rate was limited to 8 mm^3^ s^–1^. The infill density was set to 100% for all printed specimens. Cooling was disabled during the first three layers and subsequently varied between 30% and 80% according to the layer time. Additional forced cooling was applied during bridge and overhang deposition. Retraction parameters were set to 0.8 mm at 30 mm s^–1^, combined with a Z-hop height of 0.4 mm and a minimum travel distance of 2 mm.

Dimensional calibration procedures for FFF-printed specimens have been previously described by Nascimento et al.,^32^ who used nominal 20 mm calibration cubes to estimate shrinkage compensation factors.

### 2.5 Postprocessing of Samples

FTIR measurements were carried out directly on the extruded filaments so that the spectra reflected the chemical features of the materials after melt processing. For the remaining characterizations, the extruded filaments were chopped into pellets using a rotary cutter (AX Plásticos, São Paulo, Brazil). These pellets were used directly for rheological measurements and subsequently processed by injection molding to obtain mechanical test specimens for tensile, impact, and DMA analyses. All postprocessing steps were conducted under controlled laboratory conditions at 23 ± 2 °C and relative humidity of 60 ± 5%. For SEM analysis, fracture surfaces obtained after cryogenic fracture were stored in dry, labeled containers until characterization.

### 2.6 Characterization Methods

#### 2.6.1 Fourier Transform Infrared (FTIR) Spectroscopy

FTIR spectra were collected using an ATR PRO450-S infrared spectrometer (JASCO Corporation, Tokyo, Japan). Measurements were performed over the 4000–500 cm^–1^ range, with a spectral resolution of 4 cm^–1^ and 32 scans per sample. The spectra of the polymer-based formulations were baseline-corrected and normalized using the CH_2_ asymmetric stretching band at approximately 2916 cm^–1^ as an internal reference.

#### 2.6.2 Scanning Electron Microscopy (SEM)

The fracture morphology of the blends was examined by SEM using a TESCAN MIRA3 microscope (TESCAN, Brno, Czech Republic). Prior to observation, the specimens were fractured in liquid nitrogen to obtain fresh internal surfaces representative of the material microstructure. The fractured surfaces were then sputter-coated with a thin layer of gold for 120 s to minimize charge accumulation during imaging. SEM observations were performed at an accelerating voltage of 15 kV and a working distance of approximately 15 mm. Micrographs were acquired at a magnification of 5000× to evaluate phase morphology, dispersion of the elastomeric domains, and distribution of ZnO particles within the polymer matrix. All images were obtained in secondary electron (SE) mode, which provides high sensitivity to surface topography and is well suited for morphological characterization of polymeric materials. Focus, contrast, and brightness were initially adjusted automatically and subsequently refined manually when necessary to ensure image clarity and consistency among the different formulations.

For the semiquantitative morphological assessment, the embedded 10 μm scale bars were used to calibrate the micrographs. The maximum visible dimensions of clearly resolved apparent SEBS domains, pull-out cavities, and ZnO-rich agglomerates were estimated directly from the images. Dispersion uniformity was compared among the formulations based on the apparent size, relative occurrence, and spatial distribution of these features.

#### 2.6.3 Dynamic Mechanical Analysis (DMA)

DMA measurements were carried out on a Q850 DMA (TA Instruments, New Castle, DE, USA) in dual cantilever bending mode. Tests were conducted from −80 to 150 °C at a heating rate of 2 °C/min and a fixed frequency of 1 Hz. The preload and strain amplitude were adjusted according to the expected stiffness of the PP/SEBS-based blends, ensuring that the deformation remained within the linear viscoelastic regime.

#### 2.6.4 Rheological Measurements

Steady-state rheological measurements (viscosity as a function of shear rate) and oscillatory rheological analyses (*G*′ and *G*″ as functions of angular frequency) were carried out using a stress-controlled rheometer (AR-G2, TA Instruments, New Castle, DE, USA) equipped with 25 mm parallel plates and a 1.0 mm gap under a nitrogen atmosphere at 230 °C. For the oscillatory measurements, a constant strain amplitude of 1% was applied, remaining within the linear viscoelastic regime for all investigated formulations.

#### 2.6.5 Tensile Properties

Tensile tests were carried out on a universal testing machine (Shimadzu, Kyoto, Japan) equipped with mechanical grips (model MWG-5kNA, 5 kN capacity). Injection-molded specimens were tested according to ASTM D638 at a crosshead speed of 50 mm min^–1^ and room temperature (23 °C). For each formulation, five specimens were tested.

#### 2.6.6 Notched Izod Impact Testing

Pendulum impact measurements were carried out on a CEAST/Instron 9050 impact tester (Instron, Norwood, MA, USA) fitted with a 5.5 J striker. Notched Izod specimens were prepared in accordance with ASTM D256, using standard dimensions (width 10.16 mm, thickness 3.17 mm). For each formulation, five specimens were tested (*n* = 5). The absorbed energy was recorded automatically by the CEAST system, configured according to the standard, including a 150° release angle displayed on the equipment control panel.

## 3 Results and Discussion

### 3.1 FTIR Analysis

Figure 1 shows the FTIR spectra of the PP/SEBS-based blends. The neat PP/SEBS formulation exhibits the typical absorption profile of polyolefin-based systems, dominated by C–H stretching and bending vibrations, with no clear evidence of significant interfacial interactions.

**Figure 1:**
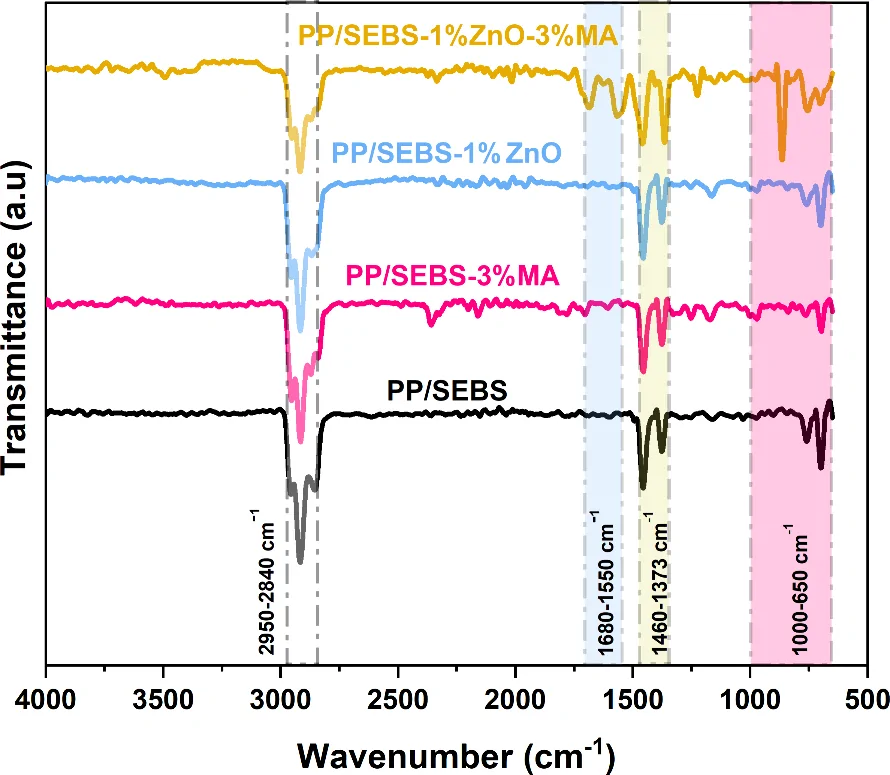
FTIR spectra of PP/SEBS-based blends. All formulations exhibit the characteristic C–H stretching bands in the 2950–2840 cm^–1^ region. The MA-containing formulations show subtle modifications in the 1680–1550 cm^–1^ region, consistent with interphase modification. The hybrid PP/SEBS-1%ZnO-3%MA system exhibits the most evident spectral changes.

The addition of ZnO alone (PP/SEBS-1%ZnO) produces only subtle spectral changes relative to neat PP/SEBS, suggesting that nanoparticle incorporation without MA does not substantially modify the interfacial chemical environment detectable by FTIR. The characteristic C–H stretching bands in the 2950–2840 cm^–1^ region remain essentially unchanged for all formulations.

By contrast, the MA-containing formulations exhibit noticeable modifications in the 1680–1550 cm^–1^ region, consistent with modifications in the interphases. These changes become more evident in the hybrid PP/SEBS-1%ZnO-3%MA formulation, which also shows differences in band shape and intensity in the 1460–1373 cm^–1^ region. Pronounced differences are also observed in the 1000–650 cm^–1^ fingerprint region, particularly for the PP/SEBS-1%ZnO-3%MA formulation, which exhibits an intensified absorption near 859 cm^–1^. This feature may reflect changes in the conformational organization of the polymer phase; however, its assignment cannot be conclusively established from FTIR alone. Taken together, these results support the view that MA modifies the blend interphase, particularly when combined with ZnO, in agreement with the improved morphology, viscoelastic dissipation, and impact performance observed for the hybrid system.

### 3.2 SEM Analysis of Cryofractured Surfaces

Representative SEM micrographs of the cryofractured surfaces of the PP/SEBS-based blends obtained after liquid-nitrogen fracture are presented in Figure 2, revealing significant morphological differences among the formulations depending on ZnO and MA incorporation.

**Figure 2:**
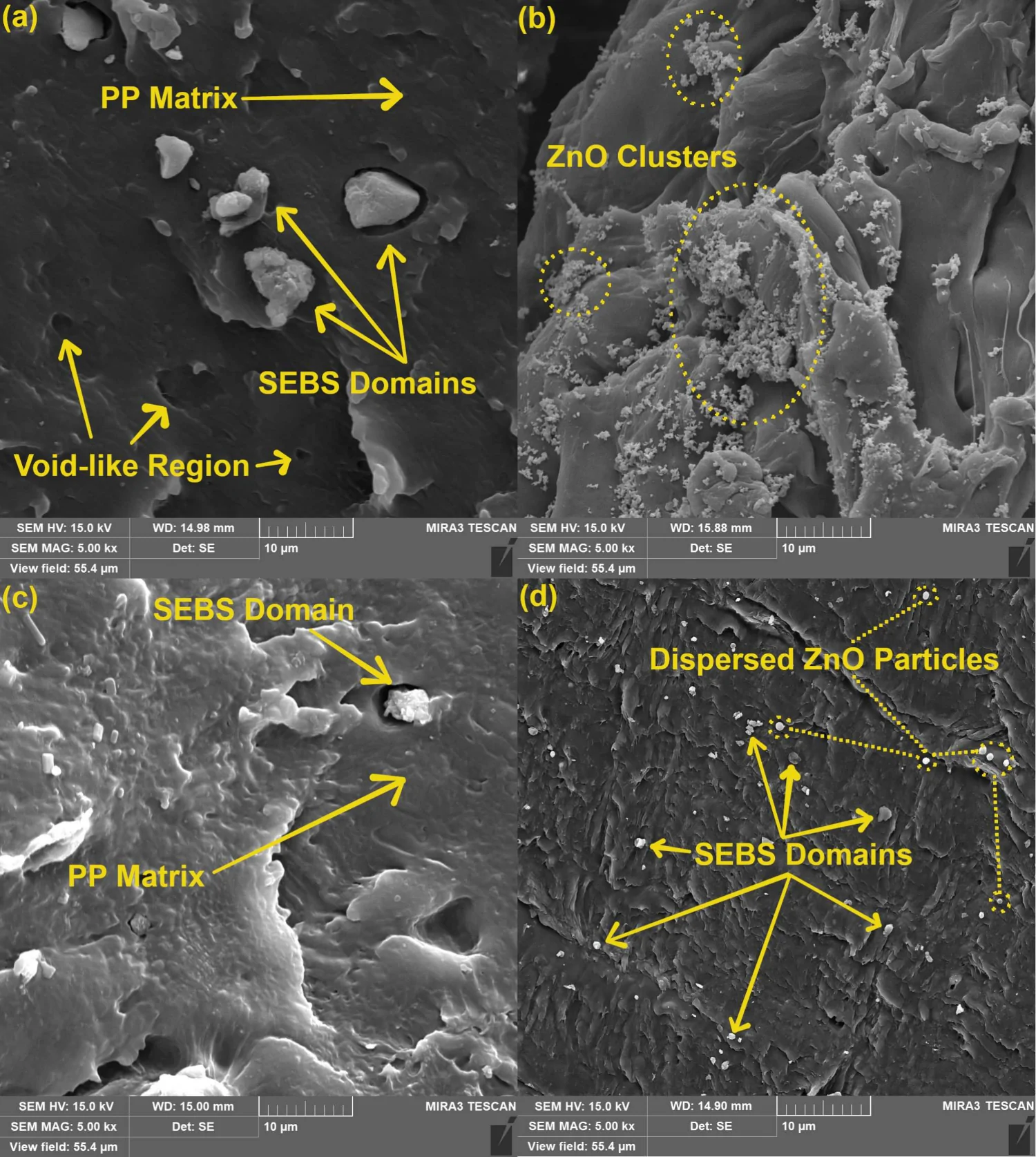
SEM micrographs of cryofractured surfaces of PP/SEBS-based blends at 5000× magnification: (a) PP/SEBS; (b) PP/SEBS-1%ZnO; (c) PP/SEBS-3%MA; (d) PP/SEBS-1%ZnO-3%MA. Arrows and circles indicate representative morphological features observed in each formulation.

In the neat PP/SEBS blend (Figure 2a), the fractured surface is relatively smooth and is characterized by a continuous PP-rich matrix containing dispersed SEBS domains. This morphology evidence the phase separation typically found in immiscible polymer blends, in which the elastomeric phase is dispersed within the thermoplastic matrix. The presence of cavity-like features and void-like regions suggests pull-out of SEBS domains during fracture, consistent with limited interphase compatibility between the blend phases. Such behavior is typical of noncompatibilized systems, in which crack propagation preferentially occurs along the polymer–polymer interface.^11,15^

After ZnO incorporation, as shown in Figure 2b, numerous bright particulate structures become visible on the fractured surface. These features, often exhibiting a cauliflower-like morphology, are attributed to ZnO-rich clusters. Their heterogeneous distribution indicates that ZnO dispersion is not fully homogeneous in the absence of MA. This behavior is expected because the high surface energy of ZnO nanoparticles favors particle–particle interactions, promoting agglomeration during melt processing.^27^

For the PP/SEBS-3%MA blend (Figure 2c), the fracture surface becomes more continuous and less marked by cavities. The polymer matrix appears more cohesive, and the SEBS domains seem more integrated into the PP-rich phase. The reduction in voids and pull-out regions is consistent with interphase modification and reduced phase separation within the blend. Accordingly, the addition of MA appears to hinder elastomer-domain pull-out during fracture. Although SEM observations alone cannot demonstrate chemical compatibilization or improved interfacial bonding, these morphological features are consistent with the complementary FTIR, rheological, DMA, and mechanical results presented in the following sections.^11,16^

Finally, in the hybrid PP/SEBS-1%ZnO-3%MA blend (Figure 2d), both dispersed ZnO particles and a more cohesive polymer morphology are observed. The ZnO particles appear more uniformly distributed over the fracture surface, indicating a more homogeneous dispersion in the polymer matrix in the presence of MA. Moreover, the surface shows greater roughness and local deformation features around the particles, suggesting improved stress transfer between the matrix and the particulate phase. The combined use of ZnO and MA promotes a more integrated microstructure, in which the improved PP/SEBS interphase facilitates a more homogeneous dispersion of ZnO particles throughout the polymer matrix.

To complement these qualitative observations, a field-based semiquantitative comparison was performed using the apparent feature dimensions estimated from the embedded scale bars. In the neat PP/SEBS blend, the clearly resolved apparent SEBS domains were approximately 4–8 μm in the displayed field, whereas smaller features of approximately 2 μm were associated mainly with pull-out cavities. PP/SEBS-1%ZnO retained coarse SEBS-related domains and contained several ZnO-rich agglomerated regions larger than 5 μm, consistent with heterogeneous filler dispersion. PP/SEBS-3%MA showed fewer cavities and a lower occurrence of coarse domains, indicating greater phase continuity. In the hybrid PP/SEBS-1%ZnO-3%MA formulation, the apparent SEBS-related domains were predominantly smaller than approximately 3 μm, while the ZnO-rich features were more finely dispersed and no large agglomerates comparable to those observed in the ZnO-only formulation were identified. This trend agrees with the quantitative results previously reported for related PP/SEBS formulations, in which the equivalent diameter of the apparent SEBS-rich domains decreased from 7.57 ± 4.79 μm in PP/SEBS to 1.34 ± 0.58 μm in the MA/ZnO-containing blend.^32^ Because the present measurements were obtained from representative secondary-electron micrographs, they should be interpreted as a semiquantitative comparison rather than as a statistically representative domain-size distribution.

### 3.3 Dynamic Mechanical Behavior

Figure 3 summarizes the temperature-dependent dynamic mechanical response of the PP/SEBS blends, reported as storage modulus (*E*′), loss modulus (*E*″), loss factor (tan δ), and a Cole–Cole-type representation (*E*″ vs *E*′).

**Figure 3:**
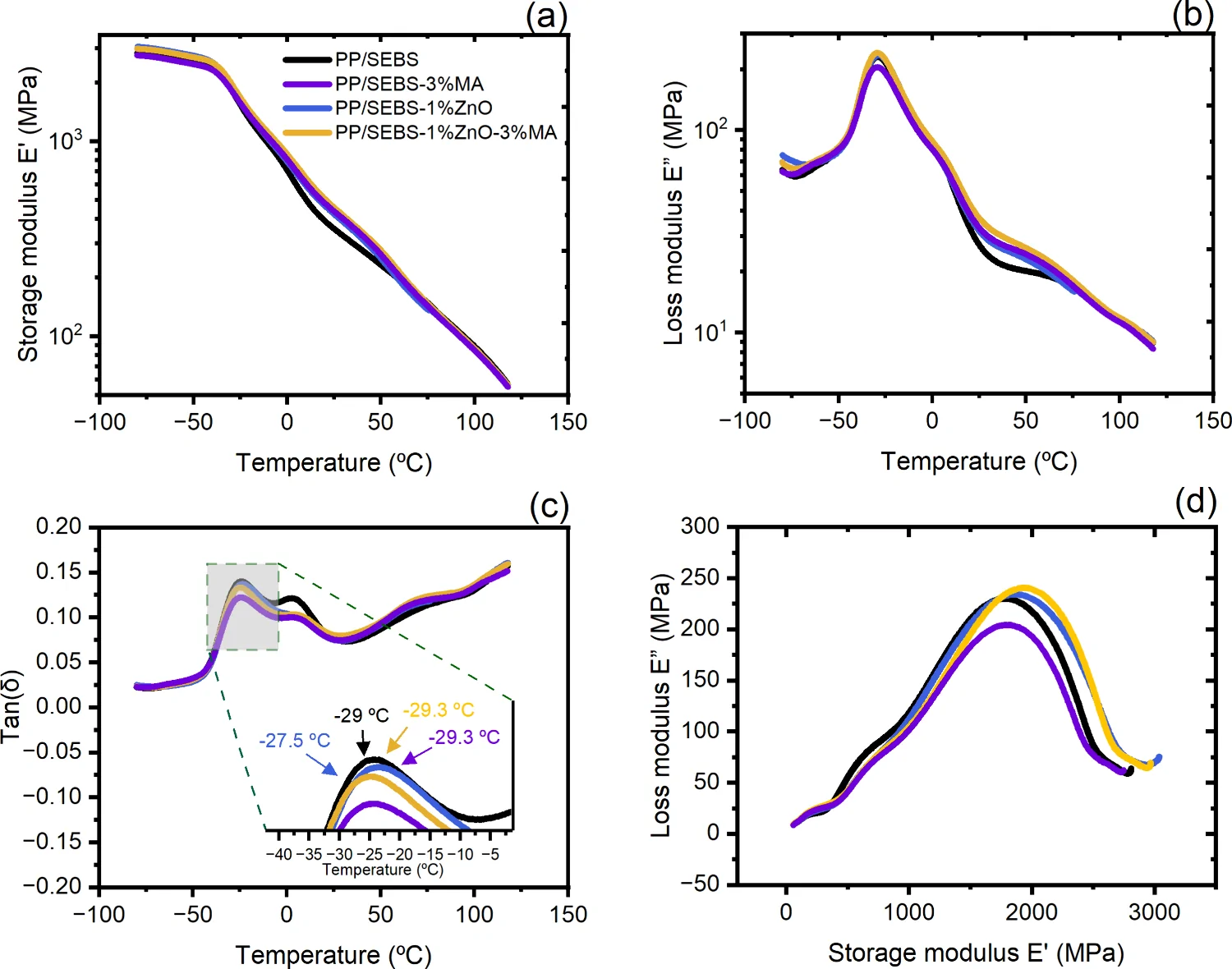
DMA of PP/SEBS-based blends: (a) storage modulus (*E*′); (b) loss modulus (*E*″). (c) Damping factor [tan­(δ)], with an inset highlighting the glass transition temperature (*T*_g_) of the SEBS phase. (d) Cole–Cole plots (*E*″ vs *E*′) correlating viscoelastic behavior with the phase structure.

As shown in Figure 3a, all formulations exhibit the expected monotonic decrease in *E*′ with increasing temperature, with a pronounced drop in the main relaxation region spanning approximately −45 to +10 °C. In this interval, *E*′ decreases from about 2600 to 3000 MPa (glassy regime, *T* ≲ −50 °C) to roughly 400–700 MPa near room temperature, corresponding to a reduction on the order of ∼70–80%.

PP/SEBS-3%MA shows a slightly reduced *E*′ across most of the subambient range. At ∼−80 °C, *E*′ is on the order of ∼2700 MPa for PP/SEBS-3%MA versus ∼2800 MPa for PP/SEBS, suggesting a mild softening effect that can be associated with changes in interfacial mobility and/or morphological refinement in this immiscible system.^6,7^

By contrast, the ZnO-containing formulations exhibit the highest dynamic stiffness at low temperatures: PP/SEBS-1%ZnO and PP/SEBS-1%ZnO-3%MA reach ∼2900–3000 MPa at ∼−80 °C, consistent with the contribution of a rigid ceramic phase and the formation of polymer–particle interactions that increase the effective reinforcing volume.^33^

The loss-modulus curves (Figure 3b) exhibit a dominant subambient dissipation peak centered at approximately −30 °C, with *E*_max_″ in the ∼200–250 MPa range depending on the formulation. The ZnO-containing blends, PP/SEBS-1%ZnO and PP/SEBS-1%ZnO-3%MA, exhibit the highest *E*″ peaks, whereas PP/SEBS-3%MA shows the lowest peak intensity. This trend indicates that the higher dissipative response is primarily associated with the presence of ZnO rather than with MA alone. The hybrid PP/SEBS-1%ZnO-3%MA formulation retains the higher dissipative response associated with ZnO while also presenting the refined morphology observed by SEM.

The damping response (Figure 3c), expressed as tan δ = *E*″/*E*′, provides a compact view of relaxation processes and energy dissipation. A small peak-position shift, which is more evident for PP/SEBS-1%ZnO (from ∼−29 to ∼−27.5 °C), indicates a slight modification in the relaxation behavior of the ZnO-containing formulation. By contrast, PP/SEBS-3%MA and PP/SEBS-1%ZnO-3%MA show only marginal changes in peak position, suggesting that MA addition predominantly influences the damping magnitude rather than the characteristic relaxation temperature.

The Cole–Cole plot (Figure 3d) exhibits a single broad arc without a clearly resolved secondary loop, indicating that the dominant relaxation processes remain coupled within the blend rather than occurring as distinct phase-specific relaxations.^34^

In addition, a clear vertical separation is observed among compositions. The ZnO-filled blends (PP/SEBS-1%ZnO and PP/SEBS-1%ZnO-3%MA) are shifted upward, exhibiting higher *E*″ at comparable *E*′, which is consistent with the higher dissipative response observed for the ZnO-containing formulations. Importantly, the hybrid PP/SEBS-1%ZnO-3%MA maintains the highest *E*″ levels across most of the arc while retaining elevated *E*′, supporting the improved balance between stiffness and energy dissipation observed for the hybrid formulation. In contrast, the MA-only formulation (PP/SEBS-3%MA) yields a depressed arc, a lower *E*″ at a given *E*′, in accordance with the lower dissipative response observed for this formulation throughout the DMA measurements.

### 3.4 Rheological Behavior

The rheological behavior of the PP/SEBS-based formulations was investigated through steady shear viscosity and oscillatory frequency sweep analyses in order to evaluate the influence of MA addition and ZnO incorporation on melt-flow behavior and viscoelastic response. These properties are particularly relevant for FFF, since extrusion-based additive manufacturing requires a balance between processability, melt flow, and structural stability after deposition.^35^

Figure 4a shows the apparent viscosity as a function of shear rate for all investigated formulations. All systems exhibited a non-Newtonian pseudoplastic behavior, characterized by a progressive viscosity reduction with increasing shear rate. Such shear-thinning behavior is highly desirable for FFF processing because it facilitates material flow through the nozzle under high shear conditions while maintaining sufficient viscosity after deposition to support dimensional stability.

**Figure 4:**
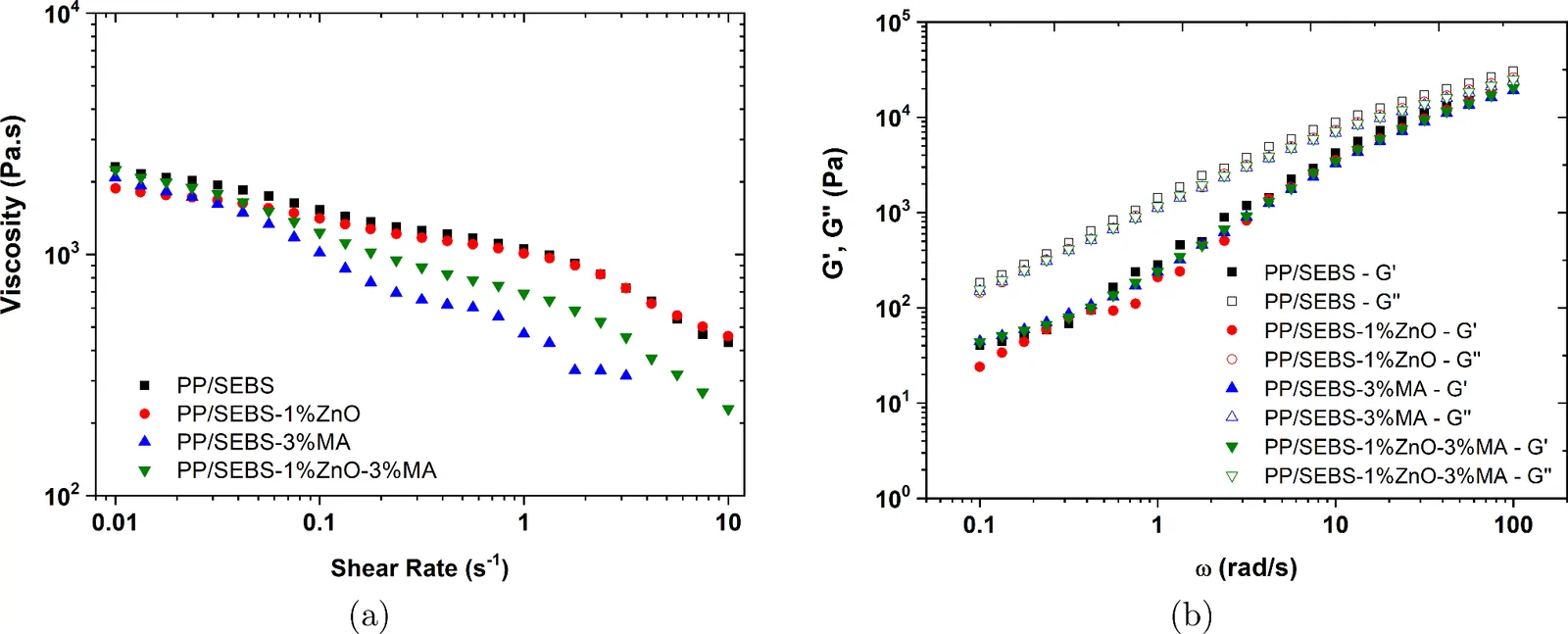
Rheological behavior of PP/SEBS-based formulations: (a) steady shear viscosity as a function of shear rate; (b) storage modulus (*G*′) and loss modulus (*G*″) as functions of angular frequency. The hybrid PP/SEBS-1%ZnO-3%MA formulation exhibited the most balanced rheological response, combining pronounced shear-thinning behavior with improved viscoelastic integrity.

The neat PP/SEBS blend exhibited the highest viscosity over most of the investigated shear-rate range, indicating greater resistance to flow during processing. The incorporation of ZnO alone (PP/SEBS-1%ZnO) produced only a slight viscosity reduction, suggesting that the nanoparticle addition did not substantially alter the melt-flow characteristics of the matrix under the investigated conditions.

In contrast, the addition of MA alone (PP/SEBS-3%MA) resulted in a pronounced viscosity decrease, particularly at intermediate and high shear rates. This behavior indicates enhanced chain mobility and reduced resistance to deformation, which may be associated with modifications in the blend interphase and partial changes in phase morphology promoted by MA. Although such a reduction may facilitate extrusion, excessively low viscosity can compromise melt strength and dimensional stability during filament deposition.

Interestingly, the hybrid PP/SEBS-1%ZnO-3%MA formulation exhibited an intermediate and more balanced rheological response. While the system maintained pronounced shear-thinning behavior, its viscosity remained consistently higher than that of the MA-only formulation at intermediate and high shear rates. This result suggests that ZnO partially compensates for the excessive viscosity reduction induced by MA, leading to a more controlled melt-flow behavior.

The oscillatory rheological response of the formulations is presented in Figure 4b, which shows the storage modulus (*G*′) and loss modulus (*G*″) as functions of angular frequency. No crossover between *G*′ and *G*″ was observed within the investigated frequency range for any formulation. Instead, *G*″ remained consistently higher than *G*′, indicating that all blends retained a predominantly viscous melt response. Although MA and ZnO modified the magnitudes of both viscoelastic moduli, they did not alter the overall viscoelastic character of the systems within the investigated frequency range.

The neat PP/SEBS blend exhibited the highest viscoelastic moduli, reflecting the greater structural resistance of the noncompatibilized matrix under oscillatory deformation. The incorporation of MA alone (PP/SEBS-3%MA) reduced both *G*′ and *G*″, indicating increased molecular mobility and lower resistance to viscoelastic deformation. This behavior is consistent with the viscosity reduction observed under steady shear conditions.

By contrast, the formulations containing ZnO exhibited higher viscoelastic moduli than the MA-only system, suggesting that the nanoparticles contribute to partial structural reinforcement of the molten matrix. In particular, the hybrid PP/SEBS-1%ZnO-3%MA formulation displayed a more balanced viscoelastic profile, maintaining higher *G*′ values than PP/SEBS-3%MA while preserving favorable flow characteristics.

Taken together, the rheological results indicate that the simultaneous incorporation of MA and ZnO produces a synergistic effect on the melt behavior of the blends. While MA improves processability by reducing viscosity and facilitating flow, ZnO contributes to preserving melt strength and viscoelastic integrity. As a result, the hybrid PP/SEBS-1%ZnO-3%MA formulation exhibited the most attractive rheological profile for FFF applications, combining efficient shear-thinning behavior, adequate flow during extrusion, and improved structural stability after deposition.

### 3.5 Mechanical Properties: Tensile and Impact Behavior

Figure 5 presents the representative engineering stress–strain curves of the investigated blends, complementing the quantitative tensile properties reported in Table 2. Besides confirming the differences in tensile strength and elongation at break, the complete stress–strain response reveals distinct postyield deformation and strain-hardening behaviors. In particular, the hybrid PP/SEBS-1%ZnO-3%MA formulation maintains higher stress over a broader strain range, corroborating its superior mechanical performance.

**Figure 5:**
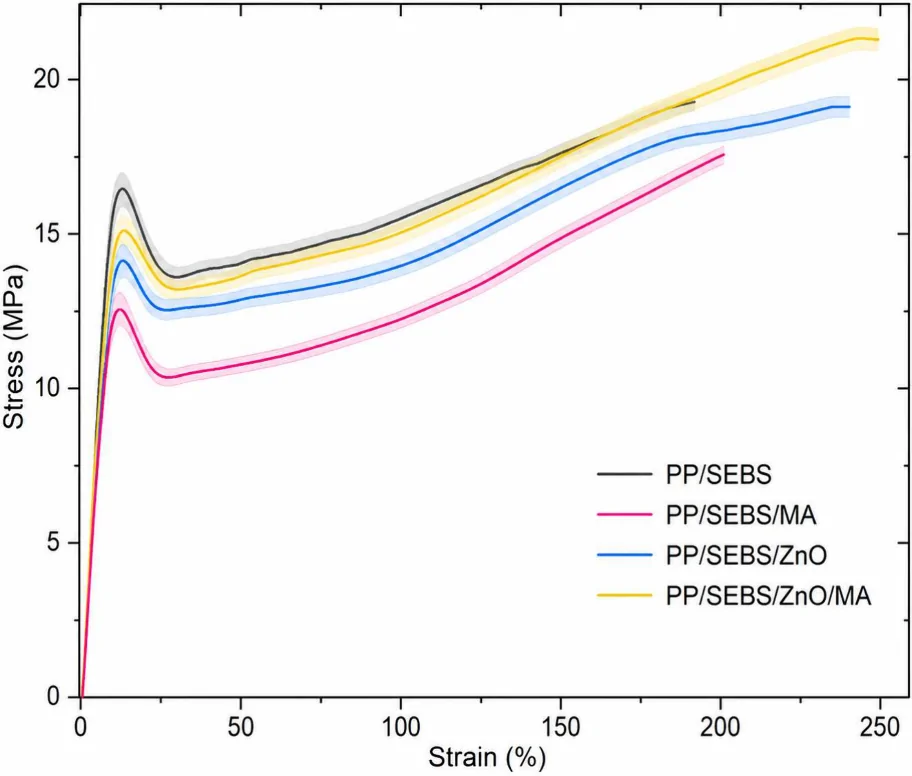
Representative tensile stress–strain curves of the investigated PP/SEBS-based blends. The shaded regions represent the experimental variability among the tested specimens.

**Table 2:** Mechanical Properties of PP/SEBS-Based Blends^a^

sample	impact (kJ m^–2^)	elongation (%)	UTS (MPa)	modulus (MPa)	*F*_max_ (N)
PP/SEBS	29.65 ± 2.61^ab^	201.93 ± 25.12^c^	18.91 ± 0.38^b^	270 ± 32^a^	287.66 ± 5.78^b^
PP/SEBS-1%ZnO	26.34 ± 3.44^b^	242.50 ± 5.11^b^	18.65 ± 1.05^b^	220 ± 11^b^	283.67 ± 15.97^b^
PP/SEBS-3%MA	32.15 ± 3.34^a^	200.00 ± 10.26^c^	18.29 ± 1.27^b^	210 ± 5^b^	278.17 ± 19.32^b^
PP/SEBS-1%ZnO-3%MA	46.86 ± 0.73^c^	265.06 ± 6.81^a^	21.11 ± 1.21^a^	270 ± 9^a^	321.08 ± 18.40^a^

a impact corresponds to impact strength, elongation corresponds to elongation at break, UTS denotes ultimate tensile strength, modulus corresponds to Young’s modulus, and *F*_max_ denotes maximum force. Values are reported as mean ± standard deviation (*n* = 5). Different superscript letters within the same column indicate statistically significant differences among formulations (one-way ANOVA followed by Tukey’s posthoc test, *p* < 0.05).

Overall, the mechanical response of the investigated systems is strongly governed by interfacial interactions and phase morphology, reinforcing that the macroscopic performance of immiscible multiphase polyolefins depends primarily on the efficiency of stress transfer and energy dissipation at the interphase rather than solely on bulk stiffness or strength.^23,36^

The neat PP/SEBS blend exhibited a balanced mechanical response, combining moderate impact strength (29.65 ± 2.61 kJ m^–2^) with high ductility, as evidenced by the elongation at break of 201.93 ± 25.12%. The tensile strength and Young’s modulus reached 18.91 ± 0.38 and 270 ± 32 MPa, respectively. Despite the relatively high elongation, the impact resistance remained limited, indicating that high ductility under quasi-static tensile loading does not necessarily translate into improved resistance under rapid notched impact conditions. Under such loading conditions, stress concentration at interfacial defects and coarse phase boundaries becomes dominant, favoring crack propagation and localized interfacial debonding rather than stable plastic deformation.^22,23^ This interpretation is consistent with the SEM results, which revealed cavity formation, pull-out regions, and limited interfacial cohesion in the uncompatibilized morphology.

The incorporation of ZnO alone (PP/SEBS-1%ZnO) did not produce statistically significant improvements in tensile strength or impact resistance relative to the neat blend. In fact, the impact strength slightly decreased to 26.34 ± 3.44 kJ m^–2^, while the tensile strength remained statistically unchanged (18.65 ± 1.05 MPa). By contrast, elongation at break increased to 242.50 ± 5.11%, suggesting enhanced deformability under tensile loading. This behavior is also evident in Figure 5, where the ZnO-containing blend extends to higher strain levels than the neat PP/SEBS while sustaining comparable tensile stresses throughout most of the deformation process. However, the reduction in Young’s modulus to 220 ± 11 MPa indicates partial softening of the system. The absence of significant impact improvement, despite the increased ductility, suggests that ZnO alone is insufficient to promote efficient stress transfer under dynamic fracture conditions. SEM analysis supports this interpretation, revealing heterogeneous ZnO dispersion and the presence of particle-rich agglomerates, which can act as localized stress concentrators and preferential crack-initiation sites.^6,27^ In this context, the reinforcing effect of ZnO remains limited by the poor interfacial coupling between the inorganic phase and the polymer matrix.

For the formulation PP/SEBS-3%MA, the impact strength increased to 32.15 ± 3.34 kJ m^–2^, while tensile strength remained statistically similar to the control (18.29 ± 1.27 MPa). The elongation at break (200.00 ± 10.26%) was comparable to the neat blend, whereas the Young’s modulus decreased to 210 ± 5 MPa. Consistently, Figure 5 shows that this formulation exhibits the lowest stress over nearly the entire deformation range, indicating that although MA refines the phase morphology, interphase modification alone is insufficient to enhance the bulk tensile response in the absence of effective particle reinforcement. Although MA alone did not produce dramatic improvements in bulk tensile properties, the SEM micrographs revealed a substantially more cohesive and refined morphology with reduced cavity formation and improved continuity between the PP-rich matrix and the SEBS domains. These results indicate that MA primarily promotes modification of the PP/SEBS interphase, reducing phase separation and suppressing premature interfacial debonding.^11,16^ The improved ZnO dispersion observed in the hybrid formulation is therefore interpreted as an indirect consequence of this refined polymer interphase. Such interphase stabilization is particularly important because it creates the structural conditions necessary for more efficient stress transfer and activation of localized plastic deformation mechanisms under dynamic loading.

The most significant improvement was observed for the hybrid formulation PP/SEBS-1%ZnO-3%MA, which exhibited a marked increase in impact strength, reaching 46.86 ± 0.73 kJ m^–2^, forming a statistically distinct group relative to all other systems. The corresponding stress–strain curves shown in Figure 5 demonstrate that this improvement is not restricted to the ultimate tensile properties. After yielding, the hybrid blend maintains higher stress throughout most of the deformation process, exhibiting a more pronounced strain-hardening behavior than the other formulations. This response indicates that the material efficiently transfers stress while sustaining plastic deformation over a wider strain interval before fracture.

Simultaneously, the formulation exhibited the highest elongation at break (265.06 ± 6.81%), highest tensile strength (21.11 ± 1.21 MPa), and highest maximum force (321.08 ± 18.40 N), while maintaining a Young’s modulus comparable to the neat PP/SEBS blend (270 ± 9 MPa). This simultaneous increase in impact resistance, tensile strength, and ductility is particularly relevant because improvements in toughness are commonly accompanied by reductions in stiffness or strength in elastomer-modified polyolefin systems. The preservation of modulus together with enhanced deformability indicates that the hybrid formulation promotes a more efficient balance between stress transfer and localized plastic deformation.

Importantly, neither ZnO nor MA alone produced such behavior, evidencing a clear synergistic effect when both additives are combined. SEM analysis corroborates this interpretation, showing a more homogeneous morphology with reduced interfacial voids, improved particle distribution, and more pronounced deformation features surrounding the dispersed domains. FTIR analysis further supports interphase modification in the hybrid system, while DMA results revealed enhanced viscoelastic dissipation for the ZnO-containing formulations, particularly through increased loss modulus (*E*″). In addition, the rheological analyses demonstrated that the hybrid formulation exhibited the most balanced melt behavior among the investigated systems, combining pronounced shear-thinning behavior with improved viscoelastic integrity. Unlike the MA-only system, which showed excessive viscosity reduction, the simultaneous incorporation of ZnO partially preserved melt strength and structural resistance during deformation. Such behavior is particularly advantageous for FFF, where efficient nozzle flow must coexist with adequate dimensional stability after deposition.

Taken together, the stress–strain curves presented in Figure 5, together with the quantitative mechanical data summarized in Table 2, demonstrate that the mechanical performance of the investigated blends is governed predominantly by interphase-controlled deformation and fracture mechanisms rather than by bulk reinforcement alone. The combined use of ZnO and MA promotes a transition from premature interfacial debonding toward a more stable energy-dissipation regime characterized by improved stress transfer, localized shear yielding, and enhanced plastic deformation under rapid loading conditions.^13,37,38^ Consequently, the PP/SEBS-1%ZnO-3%MA formulation exhibited the most attractive overall combination of impact resistance, ductility, tensile strength, and rheological processability, making it the most promising candidate for extrusion-based additive manufacturing applications.

### 3.6 FFF Performance

Based on this overall balance of impact resistance, ductility, tensile strength, and melt processability, the PP/SEBS-1%ZnO-3%MA formulation was selected for preliminary FFF validation. Figure 6 shows the printed calibration geometry containing internal hexagonal features with progressively reduced characteristic dimensions, together with representative measurements performed using an analog vernier caliper.

**Figure 6:**
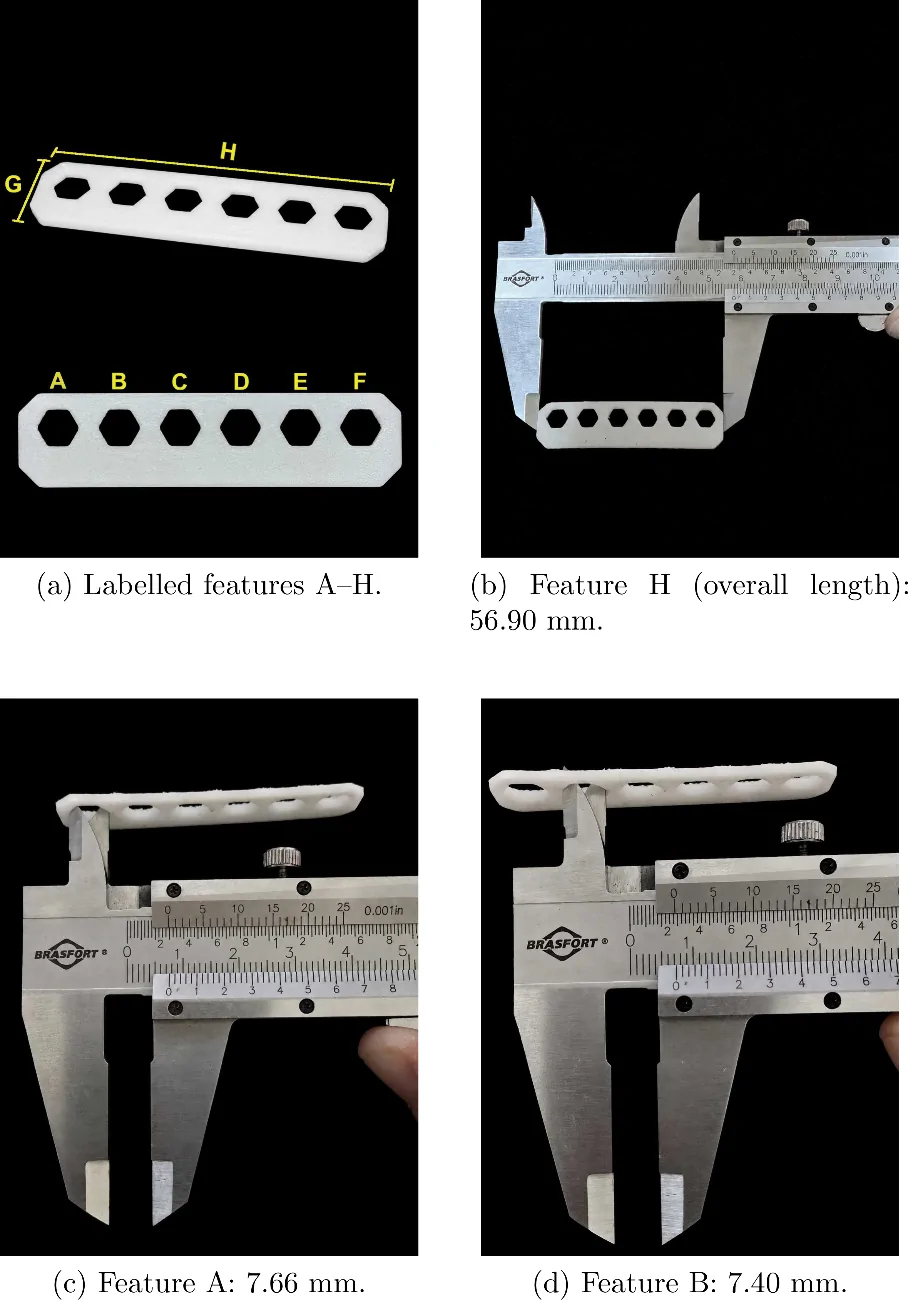
Dimensional evaluation of the FFF-printed calibration geometry containing internal hexagonal features. Panel a identifies the measured features, while panels b–d show representative vernier caliper measurements after process calibration. Nominal dimensions, measured values, and absolute deviations are reported in Table 3.

The printed specimen exhibited continuous extrusion, stable layer deposition, and satisfactory geometric preservation, without visible filament breakage or severe warping during printing. The dimensional comparison between the nominal CAD geometry and the measured printed features is summarized in Table 3.

**Table 3:** Comparison between Nominal Dimensions from the Technical Drawing and Measured Dimensions Obtained with an Analog Vernier Caliper for the FFF-Printed Calibration Geometry

feature	measured (mm)	nominal (mm)	absolute deviation (mm)
A	7.66	7.81	0.15
B	7.40	7.62	0.22
C	7.22	7.39	0.17
D	6.98	7.16	0.18
E	6.84	7.04	0.20
F	6.72	6.93	0.21
G	14.10	13.96	0.14
H	56.90	56.94	0.04

The external dimensions exhibited the lowest deviations, with the overall length (feature H) presenting an absolute error of only 0.04 mm and the overall height (feature G) showing a deviation of 0.14 mm. These results indicate good macroscopic dimensional stability and demonstrate that the selected processing parameters were sufficient to preserve the global geometry of the printed structure.

For the internal hexagonal openings (features A–F), the absolute deviations ranged from 0.15 to 0.22 mm. These values are comparable to the practical dimensional tolerances commonly reported for well-adjusted FFF/FDM systems.^39^ Considering that the investigated PP/SEBS-1%ZnO-3%MA formulation corresponds to a novel filament composition, the results indicate that the proposed material can achieve dimensional stability compatible with conventional FFF processing conditions.

Features A, C, D, and E remained within the reference deviation range of approximately 0.20 mm, whereas features B and F exceeded this value only marginally, by 0.02 and 0.01 mm, respectively. Therefore, the dimensional variations observed for the smaller internal openings remain compatible with the expected behavior of extrusion-based additive manufacturing systems, particularly for confined geometries.

The slightly higher deviations observed for some internal features can be associated with intrinsic characteristics of the FFF process, including melt spreading, local overextrusion, thermal accumulation, and the limited ability to accurately reproduce confined internal regions. These effects may become more relevant as the feature size decreases, because the deposited filament width can represent a larger fraction of the nominal geometry. However, this tendency is not strictly monotonic and depends on the combined influence of material flow behavior, toolpath generation, cooling conditions, and local geometry. Furthermore, the flow ratio adopted to improve deposition continuity and interlayer adhesion may have contributed to a slight reduction in the effective internal dimensions, although this effect cannot be isolated from the other processing variables.

Despite these local variations, the printed specimen remained structurally coherent and dimensionally consistent, indicating that the PP/SEBS-1%ZnO-3%MA formulation possesses adequate melt-processability for preliminary FFF applications. This dimensional behavior is also consistent with the rheological results discussed previously, particularly for the MA-containing formulations, which exhibited improved flow stability and a more balanced viscoelastic response during extrusion and deposition. Overall, the results demonstrate that the proposed interphase engineering strategy supports the fabrication of PP/SEBS-based filaments capable of producing printed parts with satisfactory dimensional fidelity under practical FFF conditions.

## 4 Conclusions

This study clarified the individual and combined roles of MA and ZnO incorporation in PP/SEBS blends formulated as high-performance FFF filament feedstocks. FTIR showed that ZnO alone produces only subtle spectral changes, whereas MA-containing formulations exhibit modifications consistent with interphase modification, particularly in the hybrid MA+ZnO system. This interpretation is supported by SEM of cryofractured surfaces, which showed that the uncompatibilized PP/SEBS blend exhibits coarse SEBS domains and interfacial gaps, whereas adding ZnO without compatibilization is associated with particle clusters and adjacent voids, consistent with localized stress-concentration sites. In contrast, MA promotes a finer, more cohesive phase structure, and the hybrid MA+ZnO formulation displays the most refined, deformation-rich fracture morphology with fewer debonding features, supporting a more effective energy-dissipation pathway under notched impact loading.

DMA confirmed the expected temperature-dependent softening for all compositions and indicated that ZnO-containing blends retain higher low-temperature storage modulus and exhibit enhanced subambient viscoelastic dissipation, consistent with a rigid, constrained-mobility interphase contribution. In quasi-static tension, the variations in tensile strength and elongation at break were less pronounced than those observed under notched impact loading, indicating that the main effect of MA/ZnO incorporation was associated with dynamic fracture resistance rather than with bulk tensile reinforcement alone. In contrast, notched Izod impact performance was strongly formulation-dependent: the ZnO-only blend exhibited the lowest performance, the MA-containing blend showed improved behavior relative to ZnO, and the base PP/SEBS remained intermediate, while PP/SEBS-1%ZnO-3%MA reached 46.86 ± 0.73 kJ m^–2^, representing an increase of ∼60% over the control and a statistically significant improvement.

Importantly, neither ZnO nor MA alone produced a statistically superior impact performance compared to the base blend, whereas their combined use resulted in a distinct and higher statistical group, evidencing a clear synergistic effect driven by interphase engineering.

Overall, PP/SEBS-1%ZnO-3%MA emerges as the best-performing blend by delivering the highest, statistically validated impact resistance while maintaining tensile strength and ductility. This hybrid formulation therefore represents a promising PP-based filament platform for FFF, enabling the fabrication of damage-tolerant printed components with improved resistance to handling, vibration, and accidental drops. Although the present study was limited to a single MA/ZnO composition and preliminary FFF validation, the results demonstrate the potential of interphase engineering for developing high-performance PP-based filaments. Future work should investigate different MA and ZnO contents, long-term durability, and application-specific performance to further optimize these materials for industrial additive manufacturing.

## Data Availability

All data supporting the findings of this study are provided within the article. Additional raw data (including original instrument outputs and analysis files) are available from the corresponding author upon reasonable request.

## Abbreviations

ANOVA: analysis of variance
ASTM: ASTM International
DMA: dynamic mechanical analysis
*E*′: storage modulus
*E*″: loss modulus
FFF: fused filament fabrication
*F*_max_: maximum force
FTIR: Fourier transform infrared spectroscopy
*G*′: storage modulus
*G*″: loss modulus
MA: maleic anhydride
MFI: melt flow index
PEI: poly­(ether imide)
PP: polypropylene
SE: secondary electron
SEBS: styrene–ethylene–butylene–styrene
SEM: scanning electron microscopy
tan δ: loss factor
UTS: ultimate tensile strength
ZnO: zinc oxide

## References

[1] Verma N., Awasthi P., Gupta A., Banerjee S. S. (2023). Fused Deposition
Modeling of Polyolefins: Challenges and Opportunities. Macromol. Mater. Eng..

[2] Zhou Y., Xiao S., Li H., Wang C., Li F., Liu G., Chen Z. (2025). Study on the
rheological properties and printability
of multi-scale material modified mortar for 3D printing. Construction and Building Materials.

[3] Ghanta T. S., Aparna S., Verma N., Purnima D. (2020). Review on nano-and
microfiller-based polyamide 6 hybrid composite: Effect on mechanical
properties and morphology. Polym. Eng. Sci..

[4] Kechagias J. D. (2024). Effects
of Thermomechanical Parameters on Surface Texture in Filament Materials
Extrusion: Outlook and Trends. F1000Research.

[5] Vidakis N., Petousis M., Velidakis E., Tzounis L., Mountakis N., Kechagias J., Grammatikos S. (2021). Optimization of the filler concentration
on fused filament fabrication 3D printed polypropylene nanocomposites. Materials.

[6] Murthe S. S., Sreekantan S., Mydin R. B. S. M. N. (2022). Study on the Physical, Thermal and
Mechanical Properties of SEBS/PP (Styrene-Ethylene-Butylene-Styrene/Polypropylene)
Blend as a Medical Fluid Bag. Polymers.

[7] Banerjee S. S., Burbine S., Kodihalli
Shivaprakash N., Mead J. (2019). 3D-Printable
PP/SEBS Thermoplastic Elastomeric Blends: Preparation and Properties. Polymers.

[8] Song L., Cong F., Wang W., Ren J., Chi W., Yang B., Zhang Q., Li Y., Li X., Wang Y. (2023). The Effect of Functionalized SEBS on the Properties
of PP/SEBS Blends. Polymers.

[9] Melo
de Lima L. R., Trindade T., Oliveira J. M. (2025). Effect of graphene
nanoplatelets on the properties of poly­(propylene)-based nanocomposites
for automotive applications. U.Porto Journal
of Engineering.

[10] Luna C. B. B., Siqueira D. D., Ferreira E. d. S. B., Silva W. A. d., Nogueira J. A. d. S., Araújo E. M. (2020). From Disposal
to Technological Potential:
Reuse of Polypropylene Waste from Industrial Containers as a Polystyrene
Impact Modifier. Sustainability.

[11] Luna C. B. B., Siqueira D. D., Araujo E. M., do Nascimento E. P., da Costa Agra de Melo J. B. (2022). Evaluation of the
SEBS copolymer
in the compatibility of PP/ABS blends. Polym.
Adv. Technol..

[12] Chiu H.-T., Hsiao Y.-K. (2006). Compatibilization
of Poly­(ethylene terephthalate)/Polypropylene
Blends with Maleic Anhydride Grafted Polyethylene-Octene Elastomer. Journal of Polymer Research.

[13] Hajibabazadeh S., Razavi Aghjeh M. K., Mehrabi Mazidi M. (2024). Processing–microstructure–fracture
toughness relationships in PP/EPDM/SiO2 blend-nanocomposites: Effect
of mixing sequence. Polym. Eng. Sci..

[14] Joynal
Abedin F. N., Fizal A. N. S., Alkarkhi A. F. M., Khalil N. A., Ahmad Yahaya A. N., Hossain M. S., Safie S. I., Ismail N. A., Zulkifli M. (2024). Synergistic Reinforcement with SEBS-g-MAH for Enhanced
Thermal Stability and Processability in GO/rGO-Filled PC/ABS Composites. Polymers.

[15] Wilkinson A. N., Clemens M. L., Harding V. M. (2004). The effects
of SEBS-g-maleic anhydride
reaction on the morphology and properties of polypropylene/PA6/SEBS
ternary blends. Polymer.

[16] Lima T. N., de Macedo T. C. P., Ueki M. M. (2022). Effect of sequence of melt mixing
on the properties and morphology of blends of polypropylene, styrene–ethylene–butylene–styrene
copolymer grafted with maleic anhydride, and organophilic montmorillonite
clay. Journal of Thermoplastic Composite Materials.

[17] Graziano A., Titton Dias O. A., Sena Maia B., Li J. (2021). Enhancing the mechanical,
morphological, and rheological behavior of polyethylene/polypropylene
blends with maleic anhydride-grafted polyethylene. Polym. Eng. Sci..

[18] Araujo L. M. G., Morales A. R. (2018). Compatibilization of recycled polypropylene and recycled
poly­(ethylene terephthalate) blends with SEBS-g-MA. Polímeros.

[19] Ashrith H. S., Jeevan T. P., Xu J. (2023). A Review on the Fabrication and Mechanical
Characterization of Fibrous Composites for Engineering Applications. Journal of Composites Science.

[20] Rosales C. S., Aranburu N., Otaegi I., Pettarin V., Bernal C. R., Müller A. J., Guerrica-Echevarría G. (2022). Improving the Mechanical
Performance of LDPE/PP Blends through Microfibrillation. ACS Applied Polymer Materials.

[21] Wang X., Hu S., Guo Y., Li G., Xu R. (2019). Toughened High-Flow
Polypropylene with Polyolefin-Based Elastomers. Polymers.

[22] Jia E., Zhao S., Shangguan Y., Zheng Q. (2019). Toughening mechanism
of polypropylene blends with polymer particles in core-shell structure:
Equivalent rubber content effect related to core-shell interfacial
strength. Polymer.

[23] Siviour C. R., Jordan J. L. (2016). High Strain Rate Mechanics of Polymers:
A Review. Journal of Dynamic Behavior of Materials.

[24] Liu J. (2024). Preparation and Performance
Study of Polypropylene Composites Modified
with Different Functionalized SEBS and Nano-CaCO3. ACS Applied Polymer Materials.

[25] Xiao L., Liang F., Zhou Z. (2024). Effects of
compound compatibilizer
and CaTiO3 filler on the properties of polypropylene/polyphenylene
ether/glass fiber composites. Polym. Compos..

[26] Puglisi R., Scamporrino A. A., Dintcheva N. T., Filippone G., Bruno E., Scarfato P., Cerruti P., Carroccio S. C. (2023). Photo-
and Water-Degradation Phenomena of ZnO Bio-Blend Based on Poly­(lactic
acid) and Polyamide 11. Polymers.

[27] Prasert A., Sontikaew S., Sriprapai D., Chuangchote S. (2020). Polypropylene/ZnO
Nanocomposites: Mechanical Properties, Photocatalytic Dye Degradation,
and Antibacterial Property. Materials.

[28] Maurya A. K., Mahadeva R., Manik G., Patole S. P. (2023). An investigation
into performance properties of sustainable polypropylene composites
reinforced with basalt fiber and fly ash. Polym.
Compos..

[29] do
Nascimento E. P., Luna C. B. B., Ferreira E. d. S. B., dos Santos Filho E. A., Siqueira D. D., Wellen R. M. R., Araujo E. M. (2023). Extraction
of natural fibers of Catole coconut (Syagrus cearensis): Application
as reinforcing filler in polypropylene-based composites. Polym. Compos..

[30] Diwahar P., Prakalathan K., Bhuvana K. P., Senthilkumar K. (2024). Examining
the Mechanical and Thermal Properties of a Sustainable Polypropylene
Blend and Fiber-Reinforced Composite. Polymers.

[31] Kumar P., Sharan Gupta H., Singh M., Chaudhari A. S., Maurya A. K., Manik G. (2025). Mechanical,
Thermal, and Morphological
Analysis of Himalayan Agave Fiber and Graphene Oxide-Coated Fly Ash
Hybrid Polypropylene Composites. Chem. Eur.
J..

[32] do
Nascimento C. d. D., Aguzzoli C., Souza E. G., Moraes A. C. B. K., Celso F., Schmitt P. O., Cordeiro L. A., Langone F., Rodrigues M. B. B., Tholozan L. V., Carreno N. L. V., Missio A. L., de Oliveira A. D. (2026). Development and Characterization of ZnO-Filled and
Maleic Anhydride-Compatibilized PP/SEBS Blends for 3D-Printing Filaments. J. Appl. Polym. Sci..

[33] Bashir M. A. (2021). Use of
Dynamic Mechanical Analysis (DMA) for Characterizing Interfacial Interactions
in Filled Polymers. Solids.

[34] Codou A., Anstey A., Misra M., Mohanty A. K. (2018). Novel compatibilized
nylon-based ternary blends with polypropylene and poly­(lactic acid):
morphology evolution and rheological behaviour. RSC Adv..

[35] de
Melo C. C. N., das Nascimento C. D.
d., Souza E. G., Caixeta A. L., Beatrice C. A. G., Pessan L. A., de Oliveira A. D., Machado F. M. (2026). Rheology–Performance Framework for PA6/GO Nanocomposites
via a Hybrid Processing Route. Polym. Eng. Sci..

[36] Yan J., Wang C., Zhang T., Xiao Z., Xie X. (2024). Super Tough
PA6/PP/ABS/SEBS Blends Compatibilized by a Combination of Multi-Phase
Compatibilizers. Materials.

[37] Mehrabi-Mazidi M., Razavi-Aghjeh M. K. (2021). Microscopic Deformation Behavior and Crack Resistance
Mechanism of Core–Shell Structures in Highly-Toughened PP/PA6/EPDM-g-MA
Ternary Blends. Macromol. Mater. Eng..

[38] Guo W., Zheng Z., Li W., Li H., Zeng F., Mao H. (2023). The Cellular Structure and Toughness
of Hydrogenated Styrene-Butadiene
Block Copolymer Reinforced Polypropylene Foams. Polymers.

[39] Equbal A., Murmu R., Kumar V., Asif Equbal M. (2024). A recent review
on advancements in dimensional accuracy in fused deposition modeling
(FDM) 3D printing. AIMS Materials Science.

